# Editorial: Biodegradable Materials

**DOI:** 10.3390/ijms151121468

**Published:** 2014-11-21

**Authors:** Carl Schaschke, Jean-Luc Audic

**Affiliations:** 1 School of Science, Engineering and Technology, Abertay University, Bell Street, Dundee, Scotland DD1 1HG, UK; 2 Ecole Nationale Supérieure de Chimie de Rennes, CNRS, UMR 6226, 11 Allée de Beaulieu, CS 50837, 35708 Rennes Cedex 7, France

**Keywords:** biodegradable, polymer, renewable, nanocomposite, filler, synthesis

## Abstract

This Special Issue “Biodegradable Materials” features research and review papers concerning recent advances on the development, synthesis, testing and characterisation of biomaterials. These biomaterials, derived from natural and renewable sources, offer a potential alternative to existing non-biodegradable materials with application to the food and biomedical industries amongst many others. In this Special Issue, the work is expanded to include the combined use of fillers that can enhance the properties of biomaterials prepared as films. The future application of these biomaterials could have an impact not only at the economic level, but also for the improvement of the environment.

## 1. Editorial

The development of natural biodegradable bio-based polymers has attracted significant and increasing scientific and industrial attention, particularly in terms of meeting the growing demand for sustainable development. Polymer materials derived from natural polymers such as proteins, polysaccharides, lipids or through synthesis from renewable resources are gaining increasing attention in an effort to replace petroleum-derived polymers with macromolecules. This Special Issue of the *International Journal of Material Science* presents the growing interest and focuses on the development of new biomaterials that contribute to recent findings in the field of biodegradable/bio-based materials and their applications in terms of testing, characterization, products and applications.

Natural fibers used as biocomposite fillers have received much interest in offering notable advantages over synthetic fibers. In addition to being of low cost, they are environmentally friendly, renewable and biodegradable, as well as having a low density. Examples include althaea, artichoke, arundo, bamboo, borassus fruit, coir, curaua, ferula, jute, kenaf, oil palm and sansevieria.

In Malaysia, noted as one of the world’s major oil palm producers, there is a considerable amount of oil palm biomass resulting from the milling process. The palm mesocarp fiber which is a lignocellulose fiber is a renewable material and is obtained at a very low cost directly from the oil palm mill process. It is usually burnt as a boiler fuel to generate steam and electricity for the palm oil mill. Yoon Yee Then *et al.* [1] have considered this as the feed material for other uses, and have successfully compounded it with thermoplastics of poly(lactic acid) (PLA), poly(butylene succinate) (PBS) or PLA/poly(caprolactone) blend to produce biodegradable biocomposites. This type of biocomposite offers the advantages of being light weight, low cost, biodegradable, and exhibits reasonable strength and stiffness. The technique reported is based on using superheated steam as a cost effective and green processing technique designed to modify the oil palm mesocarp fiber (OPMF) and to promote the adhesion between the fiber and thermoplastic. Under controlled operating conditions, biocomposites from superheated steam treated OPMFs and poly(butylene succinate) and PBS at various weight ratios were prepared using a melt blending technique. The mechanical properties and dimensional stability of the biocomposites were evaluated. The study revealed that the superheated steam increased the roughness of the fiber surface by the removal of surface impurities and hemicellulose. The tensile, flexural and impact properties, as well as dimensional stability of the biocomposites were markedly enhanced by the presence of treated OPMF. Scanning electron microscopy was used to show the improvement of interfacial adhesion between PBS and superheated steam (SHS)-treated OPMF for which the work concluded that superheated steam could potentially be used as an eco-friendly and sustainable processing method for modification of OPMF in biocomposite fabrication.

The majority of the world’s plastics are derived from non-biodegradable petroleum-based polymers. The persistence of these materials in the environment has had a profound impact far beyond their functional life in the form of pollution, litter and waste disposal problems. There is also concern about environmental issues and preservation of natural resources. This has led to stimulation of interest in biodegradable polymers based on renewable resources. Bio-based polymers offer environmentally friendly benefits in that they have the capability to degrade naturally into organic substances without releasing any toxic components. Of these, the thermoplastic polyester poly(lactic acid) (PLA) has attracted considerable attention as a result of its versatility, biocompatibility and biodegradabile characteristics. PLA is derived from renewable agricultural sources. In spite of its excellent properties, the relatively high production costs restrict the widespread use of PLA. One way around this is through the incorporation of low cost, renewable and fully degradable natural filler, such as oil palm empty fruit bunch (OPEFB) fiber, which is a waste product generated from the oil palm industry and widely abundant in Malaysia and other South East Asia countries. Marwah Rayung *et al.* [2] report the preparation of biodegradable composites from PLA and OPEFB fiber by a melt blending method which involves modifying the fiber through a bleaching treatment using hydrogen peroxide. The SEM micrographs, the bleached fiber composite was then found to show improved mechanical properties compared to untreated fiber composites due to an enhanced form of fiber/matrix interfacial adhesion. Interestingly, the fiber bleaching treatment also improved the physical appearance of the composite. The study was further extended by blending the composites with a commercially available colorant.

The incorporation of peptide blocks into a synthetic polymer has opened up new challenges in areas as diverse as nanotechnology such as biosensors, and biotechnology such as drug delivery systems, tissue engineering and implants. Poly(l-lactide) (PLLA) is a well-known polymer used in a number of commodity products, such as packaging materials and films, as well as specialized applications such as biomedical devices, including implants and drug delivery systems, as a result of its degradability properties within living environments. The monomer can be obtained from renewable resources such as starch from either corn or sugar beets. The combination of PLLA with peptide blocks should modify its stability because enzymatic degradation is required to hydrolyze the peptide bonds. Furthermore, the semicrystalline character of PLLA enables the formation of crystallites and amorphous phases depending on the processing conditions, allowing modulation of the PLLA influence on the self-assembly properties of the derived block copolymer. Marc Planellas and Jordi Puiggalí [3] report their work on hybrid materials which constitute peptides and synthetic polymers. This involved the synthesis of poly(l-lactide-*b*-l-phenylalanine) copolymers with various block lengths by sequential ring-opening polymerization of l-lactide and the *N*-carboxyanhydride of l-phenylalanine. The resulting block copolymers were characterized by NMR spectrometry, IR spectroscopy, gel permeation chromatography, MALDI-TOF and UV-Vis, revealing the successful incorporation of the polyphenylalanine (PPhe) peptide into the previously formed PLLA polymer chain. X-ray diffraction and differential scanning calorimetry (DSC) data were used to suggest that the copolymers were phase-separated in domains containing either crystalline PLLA or PPhe phases. They also noted a peculiar thermal behaviour using thermogravimetric analyses when polyphenylalanine blocks were incorporated into polylactide.

Liposomes have been used as a simple cell model and applied as drug carriers with broad clinical utility. The range of medical applications for liposome extends from chemotherapy for cancer and fungal infections to gene therapy. However, administration of dispersed liposomes to specific sites in the body remains a problem. A possible solution is to immobilize liposomes inside a hydrogel network and to inject the liposome gel to the diseased area. Studies on liposomes physically cross-linked by hydrophobically modified polymers have demonstrated the possibility of entrapping liposomes within a hydrogel matrix via the anchoring of the hydrophobic moieties of the polymer into the liposome bilayers. Hydrophobic moieties used include natural hydrophobic groups such as cholesterol or synthetic hydrocarbon chains. On the other hand, it is known that there is a significant functional difference in cells cultured on a flat layer and in a three-dimensional environment because three-dimensional cell culture is similar to *in vivo* conditions. There have consequently been numerous approaches for assembling cells employing various techniques and substrates, such as bioreactors, culture-plates and microfabricated surface-controlled cell adhesion. These, however, require special devices and techniques, and require long periods of time for assembly. Simple and rapid methods for assembling cells are therefore required. In achieving this, Tetsushi Taguchi and Yoshiaki Endo [4] have designed a novel biodegradable amphiphilic polymer that can assemble liposomes and cells. They prepared cholesteryl group-modified tilapia gelatins (Chol-T-Gltns) with various Chol contents per amino group of Gltn for the assembly of liposomes and cells. Liposomes were cross-linked by anchoring Chol groups of Chol-T-Gltns into lipid membranes. The resulting liposome gels were enzymatically degraded by addition of collagenase. Liposome gels prepared using Chol-T-Gltn with high Chol content showed slower enzymatic degradation when compared with gels prepared using Chol-T-Gltn with low Chol content. Using a hepatocyte cell line, good assembly properties were found and no cytotoxic effects after the addition of the high Chol content. In addition, the number of hepatocyte cells increased with concentration of high Chol-T-Gltns. It was concluded that Chol-T-Gltn can be successfully used as an assembling material for liposomes and various cell types. The resulting organization can then be applied to various biomedical fields, such as drug delivery systems, tissue engineering and regenerative medicine.

Nanocomposites formed from the dispersion of reinforcing nano-sized particles into a continuous polymer host has attracted attention in recent years as they can provide notable enhancements in the physical properties of nano-sized fillers. In addition to the use of nanoclays, carbon nanotubes, graphite and inorganic nanoparticles incorporated into polymer hosts, cellulose nanocrystals (CNCs) as a reinforcing material is now being considered. CNCs provide many benefits over other types of nano-sized fillers in that they are inexpensive, renewable, biocompatible and offer excellent mechanical properties. After acid hydrolysis of natural cellulose derived from a diversity of renewable sources including wood, cotton, ramie, bacteria, and tunicates, crystalline rod-like particles can be extracted.

To enhance the properties of the nanocomposites, nano-sized fillers have been incorporated into polymer matrices. Various types of inorganic nanoparticles, such as zinc oxide nanoparticles (ZnO-NPs), have attained increased attention and have been extensively utilized in a diversity of applications including functional devices, catalysts, pigments, optical materials, cosmetics, UV-absorbers, and additives in many industrial products.

Susan Azizi *et al.* [5] dispersed cellulose nanocrystals/zinc oxide (CNCs/ZnO) nanocomposites as bifunctional nano-sized fillers into poly(vinyl alcohol) (PVA) and chitosan (Cs) blend using a solvent casting method to prepare PVA/Cs/CNCs/ZnO bio-nanocomposites films. They investigated the morphology, thermal, mechanical and UV-Vis absorption properties, as well antimicrobial effects of the bio-nanocomposite films and were able to demonstrate that CNCs/ZnO were compatible with PVA/Cs and dispersed homogeneously in the polymer blend matrix. CNCs/ZnO also improved the tensile strength and modulus of PVA/Cs significantly as well as offering good UV-shielding properties. They also demonstrate that the biocomposite films offered good antibacterial activity towards *Salmonella choleraesuis* and *Staphylococcus aureus*.

Biodegradable nanocomposite materials offer considerable potential as an alternative to synthetic plastic packaging materials, particularly as food and beverage containers and for various disposable applications. Food packaging serves many purposes in preserving the quality and safety of foods during transportation and storage, as well as extending shelf-life by preventing unfavorable conditions such as spoilage microorganisms, chemical contaminants, oxygen, moisture and light. All forms of food packaging should also be able to control the loss or gain of moisture, prevent microbial contamination, act as a barrier against permeation of water vapor, oxygen, carbon dioxide and other volatile compounds, as well as having good mechanical strength, good thermal, chemical and dimensional stability, recyclability and biodegradability. The use of synthetic polymers fabricated from non-renewable fossil fuels such as polyethylene terephthalate (PET), polyvinyl chloride (PVC), polyethylene (PE) and many others are not totally recyclable and/or biodegradable, and therefore pose serious ecological problems. Biodegradable alternatives are highly desired such as poly(3-hydroxybutyrate) (PHB) which is a fully biodegradable and biocompatible polyester synthesized by bacterial fermentation from renewable resources such as cane sugar. However, its high crystallinity causes inherent brittleness and poor impact resistance, as well as a relatively high water vapor permeability and low resistance to thermal degradation causing the material to be easily degradable.

The use of nanofillers can improve the mechanical performance and offer a higher thermal and barrier properties. ZnO nanostructures have increasingly become the focus of considerable research due to their low cost, easy availability, biocompatibility and possibility of performing surface modifications with different functional groups. Ana M. Díez-Pascual and Angel L. Díez-Vicente [6] report the preparation of PHB-based bionanocomposites incorporating different contents of ZnO nanoparticles via a solution casting technique. This involved dispersing the nanoparticles within the biopolymer without the need for surfactants or coupling agents. As well as reporting the morphology, thermal, mechanical, barrier, migration and antibacterial properties of the nanocomposites, the nanoparticles were found to act as nucleating agents, thereby increasing the crystallization temperature and the degree of crystallinity of the matrix, and as mass transport barriers, limiting the diffusion of volatiles generated during the decomposition process, leading to higher thermal stability. Using FT-IR the Young’s modulus, tensile and impact strength of the biopolymer were improved as a result of the strong matrix-nanofiller interfacial adhesion attained via hydrogen bonding interactions. The nanocomposites exhibited reduced water uptake and improved gas and vapor barrier properties compared to PHB. The materials additionally improved the antibacterial activity against both Gram-positive and Gram-negative bacteria with increasing ZnO concentrations, an optimum balance between mechanical, barrier, migration and antibacterial properties being obtained at a critical ZnO concentration of 5.0 wt %. The migration levels of PHB/ZnO composites in both non-polar and polar simulants decreased with increasing nanoparticle content, and, promisingly, were notably below the current legislative limits required for food packaging materials.

Biomaterials offer great potential in many biomedical applications. Much work is now focusing on biodegradable cationic polymers for controlled gene delivery. Gene therapy offers the potential for treatment of various significant human diseases with gene defects such as cancer. A key challenge, however, is the requirement for safe and efficient gene delivery vectors. While recombinant viral vectors have been widely used in clinical gene therapy trials, their uncertain bio-safety issues such as oncogenicity, immunogenicity and cytotoxicity are major challenges towards successful clinical application. Non-viral gene delivery vectors, on the other hand, such as cationic polymers would overcome these problems.

Many cationic polymers, such as polylysine and polyethylenimine, are known to bind DNA to form polymer/DNA complexes (polyplexes) with nanoscale size and positively-charged surface charge, yielding detectable transfection activity *in vitro*. Their further clinical translation is restricted by either low transfection efficacy or high cytotoxicity after repeated administration. In developing new gene delivery vectors for cancer gene therapy, Kangkang An *et al.* [7] report the synthesis of a novel 4-arm poly(ethylene glycol)-*b*-poly(disulfide histamine) copolymer of poly(ethylene glycol) (PEG) vinyl sulfone and amine-capped poly(disulfide histamine) oligomer (4-arm PEG–SSPHIS). This copolymer was shown to condense DNA into nanoscale polyplexes with almost neutral surface charge. *In vitro* transfection experiments showed that polyplexes of 4-arm PEG–SSPHIS were capable of exerting enhanced transfection efficacy in MCF-7 and HepG2 cancer cells under acidic conditions. Intravenous administration of the polyplexes to nude mice bearing HepG2-tumor showed high transgene expression largely in tumor, rather other normal organs. Significantly, this copolymer and its polyplexes offers low cytotoxicity against the cells *in vitro* without causing death to the mice.

There is increasing interest in the use of aliphatic polyesters to solve so-called “white pollution” caused by traditional non-biodegradable polymers, as well as in their use as specialty polymers in biomedical applications. Polyesters are considered to be the most commercially competitive type of biodegradable polymers presently available. Of these, poly(alkylene dicarboxylate)s offer significant possibilities since most of them can be obtained from renewable resources. The extensive application of such polymers may not only mitigate the negative effect of non-degradable plastics on the environment but also reduce dependence on non-renewable fossil-fuel derived resources. Among them, poly(butylene succinate) (PBS) is regarded as the most significant poly(alkylene dicarboxylate). Angélica Díaz *et al.* [8] present a review of the more recent developments associated with polymers derived from diol and dicarboxylic acids and highlight current activities on the biomedical field. This includes the synthesis, biodegradation and applications of a series of polymers that cover a wide range of properties ranging from elastomeric to rigid characteristics that are suitable for applications such as hydrogels, soft tissue engineering, drug delivery systems and liquid crystals. The work notes the incorporation of aromatic units and α-amino acids since stiffness of molecular chains and intermolecular interactions can be drastically changed. The review also notes that poly(ester amide)s derived from naturally occurring amino acids offer significant possibilities as biodegradable materials for biomedical applications.

The use of biomaterials as films offers many applications particularly as oxygen barriers for food packaging. A major factor in their successful use is based on their hygroscopic characteristics. Arttu Miettinen *et al.* [9] present their work on assessing the three-dimensional microstructure of nanofibrillated cellulose films derived from Pinus radiata (Radiata Pine) kraft pulp fibers in terms of relative humidity. By analyzing the surface roughness, micro-porosity, thickness and their correlations using X-ray microtomography (X-μCT) and computerized image analysis, they were able to make comparisons from scanning electron microscopy and laser profilometry measurements. X-μCT is a non-destructive method used to obtain the three-dimensional structure of materials. Complemented by image analysis, X-μCT is used to estimate, the thickness, roughness and micro-porosity of nanofibrillar cellulose (NFC) films in a single measurement. Based on a series of films having varying amounts of 2,2,6,6-tetramethylpiperidinyl-1-oxyl (TEMPO)-mediated oxidated nanofibrils, X-μCT was concluded to be suitable for assessing the surface and bulk 3D microstructure of the cellulose films. Oxygen permeability tests were also carried out for the films for which the oxygen transmission rate of the films was attributed to the film porosity. The researchers were able to show that an increase in relative humidity resulted in a swelling of the films and caused a decrease of the oxygen barrier properties.

Hongli Li, Jiangping Chang *et al.* [10] studied preparations of films of poly(lactide-*co*-trimethylene carbonate) (P(LA–TMC)) and polylactide (PLA)/polytrimethylene carbonate (PTMC). Using scanning electron microscopy, they were able to show that by improving the content of TMC and PTMC, the lamellar structures of the films were altered. With increasing TMC monomer or PTMC content, the elongation at the break was notably improved. The water vapor permeability (WVP) increased for PLA/PTMC film with PTMC content, whereas it decreased with the P(LA–TMC) film. Thermogravimetric measurements were used to show that decomposition temperatures of the P(LA–TMC) and PLA/PTMC films decreased with increasing the TMC and PTMC content, respectively, but the processing temperature was significantly lower than the initial decomposition temperature. The work concludes that P(LA–TMC) or PLA/PTMC film can extend the shelf life of foods such as fruit as when used as packaging in supermarkets.

## 2. Conclusions

As guest editors, we would like to thank all the contributors for submitting their new findings in the huge field of biodegradable materials to this special issue of the International Journal of Molecular Sciences.
